# Preliminary findings of a longitudinal follow-up study of the paediatric population and their families during and after the coronavirus pandemic and the confinement

**DOI:** 10.1192/j.eurpsy.2021.971

**Published:** 2021-08-13

**Authors:** M. Gindt, A. Fernandez, M. Battista, A. Richez, O. Nachon, F. Askenazy

**Affiliations:** 1 Child And Adolescent Psychiatry, Hôpitaux Pédiatriques de Nice CHU Lenval, Nice, France; 2 Child And Adolescent Psychiatry, Hôpitaux pédiatriques de Nice CHU Lenval, Nice, France

**Keywords:** psychiatry, covid 19, Acute stress disorder, Paediatric

## Abstract

**Introduction:**

Pandemic are known to generate traumatic events, such as job losses or violence [1]. Several studies have shown that epidemics and related health measures (quarantine, confinement) lead to an increase of acute stress disorders (ASD), post-traumatic stress disorders (PTSD), anxiety and depression in the adult population [2]. In the pediatric population, few studies have been carried out on the psychiatric outcomes during and after epidemics and associated measures [3].

**Objectives:**

The aim of this study was to explore ASD symptoms during stay-at-home and Covid 19 pandemic and its impact on children and adolescent mental health.

**Methods:**

Sixty participants (53% girls and 47% boys; mean age= 9 years 5 months) were included in this longitudinal study [4]. The measures consist in an emergency semi-directed interview designed to assess symptoms of ASD according to the age of children.

**Results:**

Patients’ age modulated psychiatric outcomes. Children under the age of six shown more developmental regressions and more restlessness than older ones. Children from 6 to 12 years were characterized by more oppositional behaviors than adolescents. Finally, adolescents were characterized by more social isolation than younger ones. Other symptoms appear to be more stable across ages: sleep disturbance, fear behavior and somatization.

**Conclusions:**

Young children experienced more externalized symptoms (opposition and agitation) and developmental regressions than older children [5]. Thus, it appears necessary during pandemic to take into account the psychiatric consequences of confinement to reduce psychosocial long-term outcomes in particular in younger patients who appeared to develop specific and age-related psychiatric disorders.

